# Diversity of Volatile Emissions From Cork Oak: Quantity and Quality Vary Independently Across Its Range

**DOI:** 10.1002/ece3.72093

**Published:** 2025-08-31

**Authors:** Michael Staudt, Coralie Rivet, Meltem Erdogan

**Affiliations:** ^1^ CEFE, CNRS, EPHE, IRD Univ Montpellier Montpellier France

**Keywords:** chemical polymorphism, chemotypes, phylogeny, *Quercus suber*, terpenes, volatile organic compounds

## Abstract

Knowledge of the intraspecific variability of volatiles produced by plants is central for estimating their fluxes from ecosystems and for understanding their evolution in an ecological and phylogenetic context. Past studies suggested that volatile emissions from Cork oak ( *Quercus suber* L.) exhibit a high degree of qualitative and quantitative polymorphism. However, the extent of inherent emission variability across its range is not known. We investigated leaf emissions, photosynthetic, and morphological traits of 241 Cork oak seedlings from ten provenances. To minimize environmental influences, emissions were determined at 30°C and saturating light on seed‐grown saplings of similar age grown under the same conditions. All individuals, except for three apparent non‐emitters, released the same five monoterpenes at a mean rate of 2559 ± 120 ng m^−2^ s^−1^. Northern provenances tended to develop more sclerophyllous leaves with higher emission rates and lower photosynthetic rates than southern populations, resulting in significant different carbon losses by emissions. Independently, the emission composition varied discontinuously among individuals according to three distinct chemotypes, indicating inherent differences in the activity of two types of monoterpene synthases: one producing α‐, β‐pinene and sabinene, and the other limonene. Chemotype frequencies differed among provenances, particularly between South‐Eastern Mediterranean and South‐Western Atlantic provenances. Regarding leaf traits, we found no differences between chemotypes. The study confirms that Cork oak is a strong emitter of monoterpenes, the quality and quantity of which vary independently within its range. A comparison of the emission variability with that of other oak species suggests that a Pinene/sabinene chemotype is the ancestral form within the oak subgenus *Cerris*, which diversified during species radiation. This diversification is less pronounced in Cork oak than in other sympatric oaks, likely due to differential fragmentation and expansion of their ranges in the past.

## Graphical abstract

We investigated the VOC emissions of 241 cork oak seedlings from ten provenances. The northern provenances tended to develop more sclerophyllous leaves with higher emission rates and lower photosynthesis rates than the southern populations. Independently of this, the composition of emissions varied discontinuously according to three distinct chemotypes, whose frequencies differed between South‐Eastern Mediterranean and South‐Western Atlantic provenances. A comparison with literature data suggests that an ancestral chemotype of the oak subgenus Cerris diversified during clade radiations.

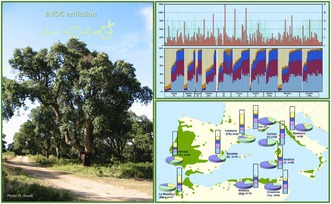

## 1 Introduction

A large number of metabolites produced by plants can evaporate under ambient temperatures, thus diffusing out of the plant organ into the air (Kesselmeier and Staudt 1999). Ecologists and environmental chemists have studied the emissions of these volatile organic compounds (VOCs) for decades, mainly for two reasons. First, the emitted VOCs can induce reactions in other receiving organisms, thereby ensuring trophic and reproductive interactions, potentially providing important benefits to the emitting species (e.g., McCormick 2016; Hirose and Satake 2024). Second, plant VOC emissions are the largest source of reactive carbon in the troposphere. Its air‐chemical breakdown shapes the oxidative capacity and radiative properties of the atmosphere, with impacts on air pollution and radiative forcing components of the climate system (Glasius and Goldstein 2016; Sporre et al. 2019; Boy et al. 2022; Liaskoni et al. 2024). To assess their impact on air quality and climate, large‐scale estimates of VOC emissions are needed (so‐called emission inventories). All of them are based on VOC emission capacities assumed for plant species or plant functional types (Monson et al. 2012).

Though some plant VOCs such as methanol are likely universally produced by all plants, others such as volatile isoprenoids, phenyl‐propanoids, or sulfuric compounds can be highly clade‐specific (Kesselmeier and Staudt 1999; Courtois et al. 2016). For example, the hemiterpene isoprene, which has been identified as the strongest contributor to global plant VOC emissions, is produced at higher rates inside the leaves of 10%–40% of the floral species depending on the study and investigated ecosystem (Fineschi et al. 2013; Sharkey and Monson 2017). Among the strongest isoprene emitters are the broadleaf trees willows, poplars, and oaks.

With about 500 species, the genus *Quercus* (oaks) is one of the most diversified genera within the Fagaceae family (Kremer and Hipp 2020). Oaks are widespread all over the Northern Hemisphere and can constitute the dominant species in temperate, subtropical, and tropical forest ecosystems. According to the latest oak phylogeny based on genetic markers, the oak genus is subdivided into two main clades, the subgenus *Quercus* and the subgenus *Cerris* (Denk et al. 2017; Hipp et al. 2020). The subgenus *Quercus* comprises five sections containing essentially but not exclusively New World oaks (sections *Protobalanus*, *Lobatae*, *Ponticae*, *Virentes*, *Quercus*) and the subgenus *Cerris* has three sections (*Cyclobalanopsis, Ilex, Cerris*) that are all Old World oaks. The oldest oak ancestors probably emerged after the Cretaceous–Paleogene extinction event (Barron et al. 2017, and references therein) and diverged into the main clades during the Eocene and Oligocene epochs. To our knowledge, all oaks of the subgenus *Quercus* analyzed for their VOC emissions have been classified as isoprene emitters due to the large dominance of isoprene in their VOC emission profiles (Figure S1). Instead, oaks of the subgenus *Cerris* are predominately either strong constitutive monoterpene (MT) emitters or non‐emitters. Furthermore, MT emitters typically produce a mixture of several isomers, the composition of which may vary depending on the species. Several previous studies have described the inter‐specific variability of constitutive VOC emissions in oaks with respect to oak phylogeny (Loreto et al. 1998; Csiky and Seufert 1999; Harley et al. 1999; Loreto 2002; Welter et al. 2012; Monson et al. 2013). For example, the deciduous North African endemic oak *Q. afares* was suspected to originate from ancient hybridization between *Q. canariensis* and *Q. suber* based on a genetic study by Mir et al. (2006). Since *Q. canariensis* belongs to the subgenus *Quercus* while *Q. suber* belongs to *Cerris*, Welter et al. (2012) hypothesized that different emitter types or dual isoprene MT emitters might occur in *Q. afares* populations as observed in early generation hybrids between other monoterpene and isoprene‐emitting oaks (Schnitzler et al. 2004; Staudt et al. 2004). The emission characteristics they found in a population of *Q. afares* resembled those of oaks of the section *Cerris* but provided no evidence for remnant isoprene production. Hence, unlike hypothesized, these results did not support the presumed hybrid origin of *Q. afares*, which was later independently confirmed by genetic studies (Simeone et al. 2018; Denk et al. 2023).

However, the VOC emissions reported in the literature for oak species and its associated assignments to an emitter type are not always consistent. For example, the Japanese oak *Q. glauca* has been described as a monoterpene‐emitting evergreen oak by Mochizuki et al. (2020), whereas it is described as a deciduous isoprene emitter by Loreto (2002). One possible reason for the inconsistency in the characterization of the interspecific variability of VOC emissions in oaks is that the species identification is not always straightforward due to the occurrence of polymorphisms associated with species migrations, repeated range fragmentations, and genetic introgression following isolated hybridization events (e.g., Simeone et al. 2018; Leroy et al. 2020). In addition, isoprenoid emissions are highly plastic and strongly vary over daytime and season, regulated by edaphic‐climatic and ontogenetic factors (e.g., Bertin et al. 1997; Llusià et al. 2013; Staudt et al. 2022). Finally, as for morphological traits, intraspecific chemical polymorphism can exist, leading to chemotypes differing inherently in the amount and/or the compositional fingerprint of VOCs, as evidenced in numerous studies for conifers and aromatic plants (e.g., Kännaste et al. 2018; Keefover‐Ring 2022).

One oak known for exhibiting potentially both qualitative and quantitative chemotypes is the Mediterranean Cork oak ( *Quercus suber* L., section *Cerris*). This emblematic evergreen oak is native to southwest Europe and northwest Africa, thriving on non‐calcareous substrates such as crystalline schists, gneiss, granite, and sands (Toumi and Lumaret 2001; Pausas et al. 2009). Mature Cork oaks can be easily and unambiguously identified by their thick cork bark. Yet, earlier works conducted on a few individuals in Italy classified Cork oak as a non‐emitter (Steinbrecher et al. 1997; Delfine et al. 2000) while studies in Spain and Portugal classified it as a strong monoterpene emitter (Pio et al. 1993, 2005). In a first study investigating the intraspecific variability in two populations in France, Staudt et al. (2004) reported that the great majority of Cork oaks are indeed MT emitters but inherently differ in their emission profiles; that is, chemotypes exist. In a follow‐up study, Loreto et al. (2009) investigated the emission variability among Cork oak saplings from five provenances and confirmed the existence of these chemotypes. However, the frequency of chemotypes and the exact compositional and quantitative variation of emissions within each Cork oak population were not reported in that study.

To gain more insight into the intraspecific variability of VOC emissions of Cork oak and its geographic distribution, we screened the VOC emissions of Cork oak saplings originating from ten provenances in Southwest Europe and Northwest Africa. VOC emission rates were quantitatively measured at defined temperature and light conditions known as standard conditions in environmental and atmospheric sciences (Niinemets et al. 2010). The resulting emission rate is referred to as the emission factor or basal emission rate (BER) and is integrated into models to predict VOC fluxes from land covers. Knowing how BER varies quantitatively and qualitatively within and between oak species will help to improve these emission inventories (Guenther 2013). It may also help to identify low‐emitting phenotypes, ecotypes, and provenances that could be used in programs of reforestation and urban greening (Sampaio et al. 2016; Morcillo et al. 2020; Chang et al. 2024). In order to minimize environmental influences on emissions, we used saplings of similar age that were grown together and kept under the same conditions during the experimental period. By focusing on the innate determinants of chemical polymorphism in VOC emissions, we aimed to answer the following questions:

Do inherent qualitative or quantitative chemotypes exist in Cork oak populations as indicated by previous studies?

What is the distribution of the putative chemotypes in the different Cork oak provenances?

Do Cork oak chemotypes or provenances differ in their foliar CO_2_/H_2_O gas exchanges or leaf morphological traits?

How chemodiversity in Cork oak compares to that of other related monoterpene‐emitting oak species and their phylogeny?

## 2 Materials and Methods

### 2.1 Plant Culture

The Cork oak acorns were collected from different trees in ten sites representing the following provenances (approximate geographic latitude, longitude coordinates): *Akfadou* Mountains in Northern Algeria (36.66, 4.62); *Catalonia* in the French Western and Spanish Northern Mediterranean (42.47, 2.92), *Corsica* island (France) around Porto Vecchio (41.63, 9.22); *Espadan* mountains in Eastern Central Spain (39.87, −0.30); *Kroumerie* Mountains in Northern Tunisia (36.79, 8.69); *Maâmora* forest region at the Northern Atlantic coast of Morocco (34.07, −6.67); *Maremma* coastal region in western central Italy north of Grosseto (42.92, 11.15); *Sardinia* island (Italy) around Iglesias (39.33, 8.53), *Setubal* district in Southwest Portugal (38.27, −8.31); *Var* department in the French Eastern Mediterranean (43.25, 6.42).

Except for the Akfadou population (see below), all plants were grown and kept in plastic pots with a mixture of argilo‐calcareous soil, sand, and peat moss compost (pH = 5,5‐6,5). Potted plants were kept outside in the institute garden, where they were irrigated during summer and occasionally fertilized. The screening experiment was run from the middle of July to the middle of October. To reduce eventual weather and seasonal effects on emissions, saplings were transferred at the beginning of July to an air‐conditioned and light‐controlled greenhouse. The daily mean incident Photosynthetic Photon Flux Density (PPFD) recorded during lighted hours ranged between 549 and 1416 μmol m^−2^ s^−1^ (mean: 1074 ± 162 SD). The mean temperature during the day and night was 23.5°C ± 1.0°C and 20.9°C ± 1.1°C, respectively.

The saplings of each provenance were randomly selected during the emission screening. To ensure that the provenances were measured at different times of the day during the experimental period, we changed the provenance after each measurement during the course of a measurement day and changed the order from 1 day to the other. To assess the plasticity of VOC emissions, we carried out four replicate measurements on five individuals during the experimental period (approximately every 3 weeks), using a different twig each time. These individuals were selected randomly from the Setubal, Catalonia, Var, Sardinia, and Maremma populations. In addition to the saplings grown in pots, we measured the VOC emissions of 18 ca. 40‐year‐old Cork oak trees of the Akfadou Mountains planted in our institute garden. To do so, we cut branches from the trees, whose cut ends were immediately immerged in a bucket with water and recut underwater to remove eventual xylem embolism. The bucket with the branch was then brought to the lab and assayed for VOC emission as described below for the potted saplings. Previous studies have shown that the leaves of cut cork oak branches emit the same constitutive VOCs in similar amounts as those of intact branches as long as they are photosynthetically active (Staudt et al. 2004).

### 2.2 VOC Emission Measurements

Foliar VOC emissions along with CO_2_/H_2_O gas exchanges were measured with a dynamic environmentally controlled exposure system consisting of two homemade flat chambers (Vol. 105 mL) run with an air flow rate of 0.7 L min^−1^ (Staudt and Lhoutellier 2011). The mature terminal leaves of a twig of a sapling were mounted in a chamber and adapted to chamber conditions for at least 30 min. Chamber air temperature was set to 30°C and incident PPFD to ca. 1500 μmol m^−2^ s^−1^. The leaves of the Cork oak are somewhat dome‐shaped and sometimes densely packed on the branches. As a result, partial self‐shading and uneven light distribution on the laminas of the enclosed leaves could not always be avoided. To ensure that photosynthesis and monoterpene synthesis still take place in the saturation light range, we used a higher PPFD value than the commonly used value of 1000 μmol m^−2^ s^−1^ (Niinemets et al. 2011).

Chamber air was delivered by a compressor (Ingersoll Rand, model 49,810,187), which was cleaned and dried by a clean air generator (Airmopure, Chromatotec, St Antoine, France) and a charcoal filter, and then re‐humidified by directing an adjustable portion of the air stream through a bypass with washing bottles. The average air to leaf vapor pressure deficit during measurements was 0.026 ± 0.005 mol mol^−1^. CO_2_ and H_2_O air concentrations of the chamber air were monitored using a LiCOR 840A infrared gas analyzer at a flow rate of 0.2 L min^−1^. VOCs were sampled by directing a portion (0.1 L min^−1^) of the chamber airs for 10 min into Perkin Elmer adsorbent cartridges containing ca. 290 mg Tenax TA. Immediately after VOC sampling, the LiCOR 840A analyzer was switched to the chamber incoming air, and the enclosed leaves were collected to determine their projected area (Delta‐T Areameter MK2, Delta‐T Devices Ltd), fresh weight, and dry weight after having dried them for 3 days at 60°C (microbalance Mettler PM200, Mettler‐Toledo SA). Empty chamber measurements were regularly conducted to detect eventual background VOCs.

Cartridges were analyzed with a Varian CP3800/Saturn2000 gas chromatograph mass spectrometer (GC–MS) equipped with a Perkin Elmer Turbomatrix thermodesorption unit. Trapped VOCs were thermally desorbed (15 min at 230°C) on a cold trap (−30°C) containing a small amount of Tenax TA, and subsequently thermally injected into a Chrompack Sil 8 CB low bleed capillary column (30 m × 0.25 mm × 0.25 μm). The GC oven temperature program was 3 min at 40°C, 3°C min^−1^ to 100°C, 2.7°C min^−1^ to 140°C, 2.4°C min^−1^ to 180°C, 6°C min^−1^ to 250°C. Carrier gas was helium (1.0 mL min^−1^). The GC–MS was calibrated with pure standards (Sigma‐Aldrich, Fluka, Roth) dissolved in methanol to realistic concentrations. For calibration, 1 μL of the standard solution was injected into the inlet of an adsorbent cartridge and immediately flushed with pure nitrogen for 10 min (flow rate approx. 50 mL min^−1^) to disperse the VOC into the adsorbent bed and expel the solvent. The repeatability of this method was better than 10% for all standards. We estimated the realistic detection limit of VOC emissions to be twice the mean VOC concentrations measured in the empty leaf chambers during the experimental period. These were between 0.2 and 0.9 ng L^−1^ for the major MTs, corresponding to pseudo‐emission rates ranging between 4 and 14 ng m^−2^ s^−1^ (approx. 40 ng m^−2^ s^−1^ for the sum of the five major MTs). In addition to the offline VOC measurements, the chambers were connected to an online gas chromatograph (AirmoVOC C2‐C6 Gas Chromatograph, Chromatotec, St Antoine, France) for the eventual detection of isoprene (see Staudt and Visnadi (2023), for a more detailed description).

### 2.3 Data Treatment and Statistics

The VOC emission rate was calculated as the difference between the VOC air concentration in the chamber enclosing leaves and the mean concentration measured in the empty chambers multiplied by chamber airflow rate, and divided by the projected leaf area or by the leaf dry mass. Further, we calculated the relative leaf water content (LWC, %) as fresh weight–dry weight/fresh weight × 100, and the specific leaf dry weight (SLW, g m^−2^) as the ratio of leaf dry mass to projected leaf area (often also referred to as LMA). The calculation of the leaf CO_2_/H_2_O gas exchange variables net‐CO_2_ assimilation (A), transpiration (E), water vapor conductance (G_H2O_), and internal CO_2_ concentration (Ci) was made according to von Caemmerer and Farquhar (1981) using the CO_2_ and H_2_O mixing ratios of the air leaving and entering the chambers. We further deduced from these data the water use efficiency (WUE = A E^−1^) and the intrinsic water use efficiency (iWUE = A G_H2O_^−1^). Variation in iWUE and Ci at given [CO_2_], light, and temperature indicates adjustments in the limitations of the leaf's efficiency to fix atmospheric CO_2_ other than stomatal opening (e.g., Rubisco activity, mesophyll conductance; see for example, Galmés et al. 2007), whereby iWUE decreases and Ci increases with decreasing efficiency. Finally, we calculated the ratio between VOC emission and A as the percent mole of the actually assimilated carbon lost by emission (C‐loss). The formation of constitutive monoterpenes and isoprene inside chloroplasts is directly linked to photosynthetic processes delivering the basic carbon substrates and energetic cofactors for the chloroplast isoprenoid biosynthesis pathway. Labelling studies with ^13^CO_2_ have shown that the major fraction, if not all, of their carbon comes from the ongoing CO_2_ fixation in the Calvin‐Benson‐Bassham Cycle (e.g., Loreto et al. 1996; for review see for example, Lantz et al. 2019). Thus, the C‐loss reflects the actual percent allocation of photosynthates to volatile isoprenoid production in chloroplasts. Given this link, we discarded all measurements from the data set in which A was very low (ca. < 1 μmol m^−2^ s^−1^) in order to avoid identifying false non‐emitters.

We analyzed relative emission data (percentage composition) with hierarchical cluster analysis and factorial discriminant analysis to differentiate chemotypes. Correspondence analyses based on Chi‐square distances were applied to the contingency table of the frequency of chemotypes in the ten provenances to test for differences in their distributions. Subsequently, we applied a Mantel test to test the correlation between the dissimilarity in chemotype frequency among provenances and their geographical distances.

To test whether provenances and chemotypes differed in total VOC emission rates (BER), CO_2_/H_2_O gas exchange variables (A, G_H2O_, Ci, WUE, iWUE, C‐loss) and leaf morphological traits (SLW, LWC, leaf size) we ran ANOVAs or Kruskal Wallis tests in case data did not fill the necessary requirements. Differences between groups were considered to be significant at the level *α* = 0.05. Posthoc Bonferroni and Dunn tests were used for pairwise comparison of categories with adjusted significance levels (*α*) of 0.0011 and 0.0083 for provenance and chemotype, respectively. Pearson correlation analyses and scatter plots were performed to test the covariation among variables. All statistical analyses were done with Addinsoft XLSTAT statistical and data analysis solution.

Unless otherwise stated, the degree of data scattering (variances) of mean values are reported as ± standard error (SE = standard deviation (SD) divided by the square root of the number of replicates) and as relative variation coefficient (VC = standard deviation divided by the mean × 100).

## 3 Results

Of 241 individuals, only three were found to be non‐ or extremely low constitutive VOC emitters. The average total VOC emission rate with these non‐emitters included was 2655 ± 121 (VC: 71%) ng m^−2^ s^−1^ or 55.8 ± 2.4 μg g^−1^ h^−1^ (VC: 68%) and the apparent photosynthetic carbon investment for VOC emissions (C‐loss) 2.3% ± 0.2% (VC: 110%). Excluding the three non‐emitters (*n* = 238), the mean total emission rate was 2698 ± 122 (VC: 70%) ng m^−2^ s^−1^ or 56.5 ± 2.4 μg g^−1^ h^−1^ (VC: 66%), increasing the mean C‐loss slightly to 2.4% ± 0.2% (VC: 109%). The average emission in moles was approx. 20 nmol m^−2^ s^−1^ and in amount of carbon approx. 50 μg C g^−1^ h^−1^. The major emitted VOCs were α‐pinene, sabinene, β‐pinene, myrcene, and limonene. These five MTs were detected in all emitting trees and accounted on average for 95% of the total emission (2559 ± 120 ng m^−2^ s^−1^; 53.7 ± 2.4 μg g^−1^ h^−1^; *n* = 238). In addition, the monoterpenes, α‐thujene, α‐terpinene, 1,8‐cineol, (Z)‐ and (E)‐*β*‐ocimene, the sesquiterpenes germacrene D and *β*‐caryophyllene, and the green leaf volatiles (Z)‐3‐hexenol and (Z)‐3‐hexenyl acetate were rarely detected, mostly at trace amounts except for ocimenes, which were more frequently observed, sometimes at high rates (> 500 ng m^−2^ s^−1^). Isoprene, if any, was only occasionally detected at trace levels (< 1 ng m^−2^ s^−1^). Given the irregular, possibly induced occurrence of ocimenes and other trace compounds, we focus on the five main constitutive MTs to characterize the inherent intraspecific variation in the emission composition.

**FIGURE 1:**
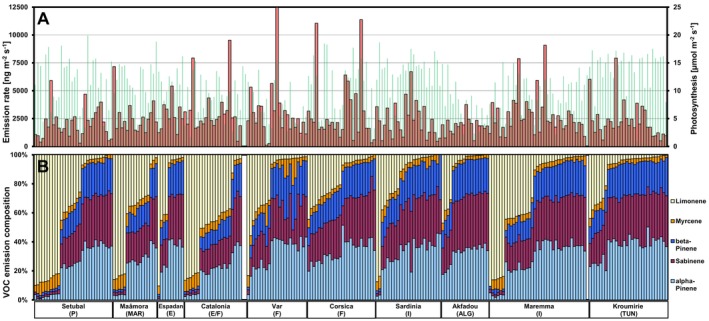
Leaf monoterpene emissions from 241 cork oak saplings originating from ten provenances. (A) Emission rate of the sum of the five main compounds (red columns) and photosynthesis rate (green columns) measured at 30°C and ca 1500 μmol m^−2^ s^−1^ incident PPFD; (B) relative proportions of the five main monoterpenes. The data were first sorted according to the origin of the trees (provenance) and then according to the proportion of limonene in their emissions. Note that three seedlings emitted no VOCs or only traces, although their leaves were fully photosynthetically active.

In all ten populations, their percentage composition varied among individuals expressing a discontinuous variation pattern (Figure 1). The emission profiles of 52.5% of the individuals consisted mainly of α‐, β‐pinene, and sabinene, whereas the profiles of 13% consisted mainly of limonene. The remaining individuals (34.5%) emitted all four compounds in high proportions. Repeated measurements conducted on five trees showed that the individual emission profiles were quite stable even though the overall quantity varied considerably within plant replicates (Figure S2). This suggests that the compositional fingerprint is largely inherent, whereas the actual production rate is more subject to plasticity even under environmentally controlled conditions. Hierarchical cluster analysis grouped the emission profiles into three clearly distinguished chemotypes as confirmed by factorial discriminant analysis (Figure S3). These chemotypes are hereafter referred to as Pinene/Sabinene type, Limonene type, and Mixed type, and the three individuals lacking these emissions as Non‐emitter. Correlation analyses of the relative emissions of single compounds show that α, β‐pinene, and sabinene scaled all positively to each other (*R*: 0.85 to 0.89) and all negatively to limonene (*R*: −0.94 to −0.97) (see Figure S4). Myrcene proportions were weakly positively correlated to limonene (*R*: 0.55) and weakly negatively to pinenes and sabinene (*R*: −0.52 to −0.68).

**FIGURE 2:**
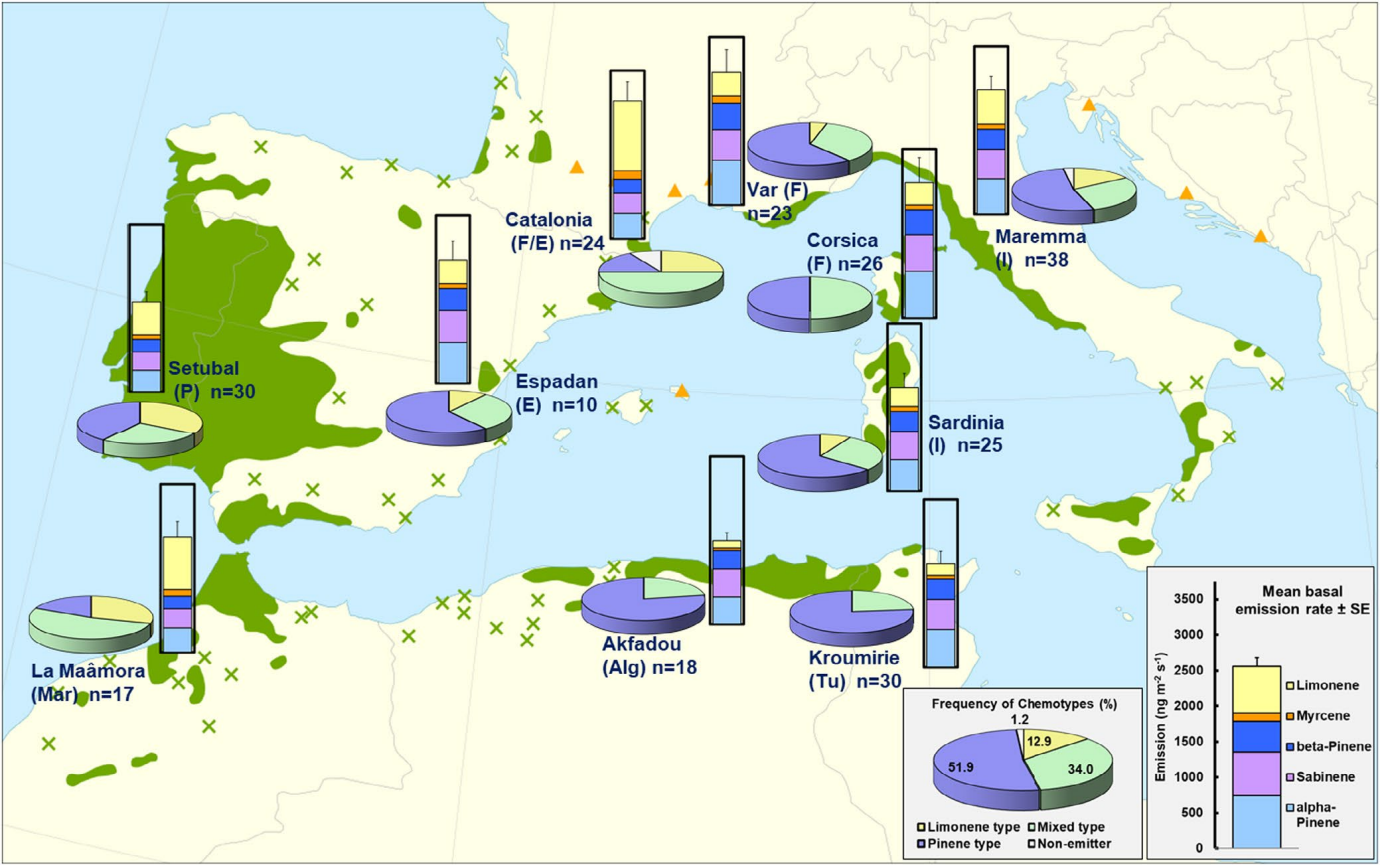
Overview of the mean emission rate (column charts) and frequency of chemotypes (pie charts) of the ten provenances within the Cork oak distribution range (*n* = number of tree replicates). In all column plots the Y‐axes are at the same scale and the error bars show the SE for the sum of the emissions of the five major monoterpenes. The big insert charts in gray show the means across all investigated provenances. The distribution map is taken from Caudullo et al. (2017). The green colored areas in the map are the continuous native Cork oak ranges and the green crosses isolated native populations. Orange triangles indicate introduced and naturalized Cork oak populations.

Based on Chi‐square statistic, correspondence analyses suggest real differences between the provenances in terms of their chemotype frequencies (*p* < 0.001). The most diverged emission profiles were between the Eastern provenances Akfadou, Kroumirie, Corsica and the Western provenances Maâmora, Catalonia and Setubal, the former group lacking completely the Limonene type, which was most present in the latter group (Figure 2). The profile of the Maremma population with the highest number of screened individuals was the closest to the mean profile of all. Results of a Mantel test suggest that the dissimilarity in the chemotype frequency among provenances is not correlated with their geographical distances (*p* = 0.053, *R* ^2^ = 0.1).

Single factor ANOVAs or equivalent Kruskal Wallis tests revealed no significant differences among chemotypes for any gas exchange or leaf morphological variable except for the extremely low mean emission rate and C‐loss of the Non‐emitter type compared to the emitting chemotypes (Table 1). In contrast, they revealed significant differences between provenances for the variables A (*p* < 0.0001), E (*p* < 0.0001), G_H2O_ (*p* < 0.0001), iWUE (*p* = 0.0001), Ci (*p* = 0.002), SLW (*p* < 0.0001), LWC (*p* < 0.0001) and leaf size (*p* < 0.0001). The provenance differences in A were essentially due to differences in G_H2O_ though not exclusively, because provenances differed also in iWUE and Ci. For example, the mean A of the Corsica saplings was not significantly lower than that of the Kroumerie saplings despite their much lower mean G_H2O_. This indicates that the leaves of the Corsica saplings fixed CO_2_ more efficiently under saturating light conditions, as evidenced by the significantly higher iWUE and lower Ci values. Provenances differed also in BER (*p* = 0.032 for the sum of major MTs) but overall in the associated photosynthetic carbon investments (C‐loss, *p* < 0.0001). On average, the northern provenances Var, Catalonia, and Maremma invested significantly more photosynthetic carbon in monoterpene production than the southern provenances, with the exception of the provenance Akfadou, which exhibited both a low mean emission and a low mean photosynthetic rate. Since measured on cut branches, its comparison with other populations should be taken with caution.

**TABLE 1:** Basal emission rates and CO_2_/H_2_O gas exchange variables of *Q. suber* saplings from 10 provenances.

	Major MT emission	Total MT emission	C‐loss (major MTs)	A	G_H2O_	Ci	WUE	iWUE	SLW	Leaf size	LWC
Provenance (*n*)	[ng m^−2^ s^−1^]	[ng m^−2^ s^−1^]	[%]	[μmol m^−2^ s^−1^]	[mmol m^−2^ s^−1^]	[ppm]	[mmol mol^−1^]	[μmol mol^−1^]	[g m^−2^]	[cm^2^ leaf^−1^]	[%]
Sardinia (25)	2320 ± 311^a^	2812 ± 359^a^	1.33 ± 0.21^a^	13.6 ± 0.6^c^	158 ± 9^cd^	205 ± 6^ab^	3.47 ± 0.14	90 ± 5^ab^	184 ± 4^a^	1.29 ± 0.07^a^	50.5 ± 0.5^ab^
Setubal (30)	2012 ± 236^a^	2051 ± 242^a^	1.70 ± 0.38^a^	11.7 ± 0.8^bc^	144 ± 11^bc^	213 ± 7^ab^	3.25 ± 0.14	85 ± 5^ab^	180 ± 4^abc^	1.38 ± 0.10^a^	52.6 ± 0.7^a^
Maâmora (17)	2572 ± 349^a^	2886 ± 393^a^	1.71 ± 0.28^ab^	12.0 ± 0.8^bc^	129 ± 7^bc^	202 ± 9^ab^	3.58 ± 0.20	94 ± 5^ab^	161 ± 4^bcd^	1.78 ± 0.14^ab^	51.3 ± 0.8^ab^
Espadan (10)	2752 ± 418^a^	2825 ± 415^a^	1.84 ± 0.33^ab^	11.8 ± 0.7^abc^	156 ± 28^bc^	207 ± 12^ab^	3.34 ± 0.22	86 ± 7^ab^	183 ± 7^ab^	1.69 ± 0.20^ab^	48.6 ± 0.8^bc^
Kroumirie (30)	2309 ± 289^a^	2322 ± 291^a^	2.03 ± 0.87^a^	13.9 ± 0.7^c^	210 ± 9^d^	229 ± 7^b^	3.37 ± 0.15	67 ± 4^a^	155 ± 4^d^	2.32 ± 0.62^bc^	53.4 ± 0.6^a^
Corsica (26)	3001 ± 560^a^	3043 ± 559^a^	2.11 ± 0.29^ab^	11.0 ± 0.9^abc^	116 ± 11^abc^	192 ± 7^a^	3.71 ± 0.17	99 ± 5^b^	175 ± 5^abcd^	1.74 ± 0.14^ab^	48.8 ± 0.5^bc^
Akfadou (18)**	1876 ± 166^a^	1889 ± 167^a^	2.32 ± 0.35^ab^	7.4 ± 0.9^ab^	99 ± 16^ab^	229 ± 8^ab^	4.21 ± 0.42	85 ± 6^ab^	157 ± 6^cd^	1.58 ± 0.24^ab^	49.3 ± 0.9^bc^
Maremma (37)	2783 ± 295^a^	2957 ± 293^a^	2.40 ± 0.25^b^	9.4 ± 0.5^ab^	114 ± 8^ab^	214 ± 6^ab^	3.45 ± 0.15	87 ± 4^ab^	169 ± 4^abcd^	2.24 ± 0.16^bc^	46.8 ± 0.4^cd^
Catalonia (24)	3065 ± 431^a^	3112 ± 430^a^	3.31 ± 0.66^b^	9.4 ± 0.8^ab^	100 ± 9^ab^	200 ± 8^ab^	3.59 ± 0.19	97 ± 6^b^	165 ± 3^abcd^	1.91 ± 0.16^ab^	48.6 ± 0.6^bc^
Var (23)	2957 ± 518^a^	3142 ± 516^a^	3.52 ± 0.58^b^	7.2 ± 0.8^a^	71 ± 7^a^	192 ± 11^a^	3.58 ± 0.20	103 ± 6^b^	187 ± 7^a^	3.37 ± 0.27^c^	45.2 ± 0.8^d^
P_Provenance_ (Test)	**0.032** (K)*	0.151 (K)*	**< 0.0001** (K)	**< 0.0001** (K)	**< 0.0001** (K)	**0.002** (K)	0.361 (K)	**0.0001** (K)	**< 0.0001** (K)	**< 0.0001** (K)	**< 0.0001 (K)**
Chemotype (*n*)											
Limonene (31)	2503 ± 314^a^	2545 ± 322^a^	2.47 ± 0.52^a^	10.8 ± 0.8	126 ± 13	208 ± 7	3.39 ± 0.16	93 ± 5	170 ± 4	1.66 ± 0.12	50.9 ± 0.8
Mixed (82)	2562 ± 197^a^	2666 ± 195^a^	2.08 ± 0.16^a^	10.7 ± 0.4	124 ± 6	207 ± 4	3.58 ± 0.10	92 ± 3	169 ± 3	1.91 ± 0.10	49.3 ± 0.5
Pinene (125)	2580 ± 173^a^	2767 ± 179^a^	2.51 ± 0.27^a^	10.8 ± 0.4	136 ± 6	211 ± 4	3.49 ± 0.09	86 ± 2	172 ± 2	2.09 ± 0.10	49.5 ± 0.4
None (3)	3 ± 1^b^	28 ± 14^b^	0.02 ± 0.01^b^	13.4 ± 0.5	139 ± 6	191 ± 21	4.32 ± 0.35	97 ± 8	177 ± 4	1.53 ± 0.26	48.3 ± 0.7
P_Chemotype_ (Test)	**0.028** (K)*	**0.023** (K)*	**0.025** (K)	0.705 (K)	0.570 (K)	0.822 (A)	0.153 (K)	0.235 (A)	0.822 (A)	0.129 (A)	0.244 (A)
Total (241)	2527 ± 120	2665 ± 123	2.21 ± 0.16	10.8 ± 0.3	131 ± 4	209 ± 3	3.53 ± 0.06	89 ± 2	171 ± 2	1.96 ± 0.06	49.6 ± 0.3

*Note:* Values are means ± SE per provenance (upper table) and per Chemotype (lower table). The line below each sub‐table summarizes the *p*‐values of ANOVA (A) or Kruskal‐Wallis (K) tests for differences between Provenance or Chemotypes (bold values are significant at *p* < 0.05). Superscript letters denote which of the categories are different based on pairwise comparisons using the Bonferroni test (A) and Dunn test (K). Note that these post hoc tests apply adjusted significance levels lower than 0.05 (for Chemotype *α* = 0.0083, for Provenance *α* = 0.0011). Therefore, the differences between pairs of categories are sometimes reported as insignificant even if the global test result is significant at *α* = 0.05.

Abbreviations: (i)WUE, (intrinsic) water use efficiency; A, CO_2_‐assimilation; Ci, Leaf internal CO_2_ mixing ratio; C‐loss, VOC emission A^−1^ in % mole carbon; G_H2O_, H_2_O‐conductance; LWC, Relative leaf water content; SLW, Specific leaf weight.

* Tests were run with emission rates expressed per leaf surface. Using emission rates expressed per leaf dry mass reduces the significance level of provenance effects for Major MT emissions (*p* = 0.092) and increases it for Total MT emissions (*p* = **0.032**). It does not change the significance level for differences between chemotypes for Major MT emissions (*p* = **0.027**) and for Total MT emissions (*p* = **0.024**).

** Measurements were made on cut branches collected from 40‐year‐old trees, which may have affected CO_2_/H_2_O gas exchanges and VOC emissions.

**FIGURE 3:**
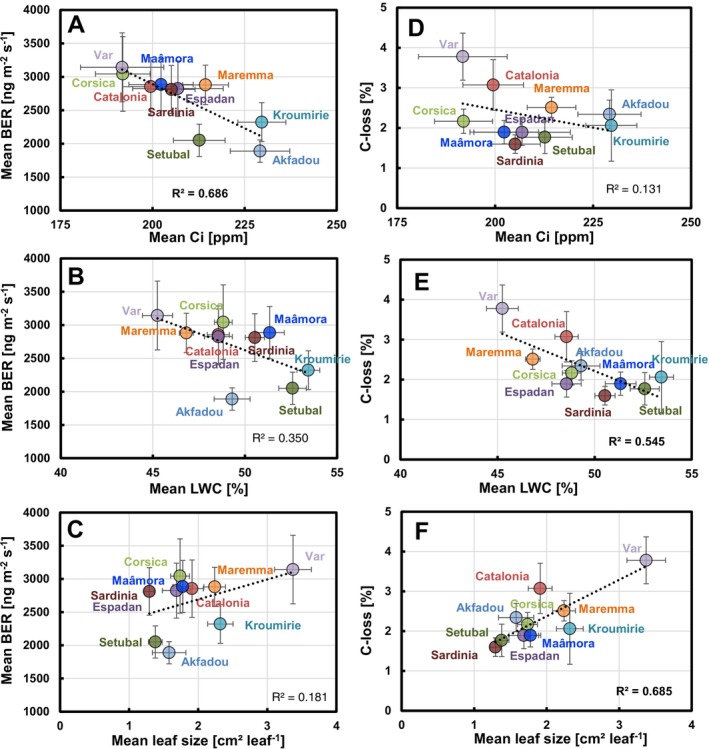
Plots of the provenance means (± SE) of the basal monoterpene emission rates (BER, left panels) and fraction of assimilated carbon lost by VOC emission (C‐losses, right panels) against leaf internal CO_2_‐concentrations Ci (A, D), relative leaf water contents (LWC, B, E) and leaf sizes (C, F). Dotted lines with coefficients of determination *R* ^2^ show results from linear correlations. Bold *R* ^2^‐values indicate significant correlations (*p* < 0.05). Correlation coefficients (*R*) and corresponding *p*‐values are summarized in the Table S1.

The provenance mean BERs per leaf surface were significantly negatively correlated with mean Ci (*R* = −0.828, *p* = 0.003) and marginally negatively correlated with mean LWC (*R* = −0.591, *p* = 0.072) (Figure 3A–C, and Table S1). They also scaled positively with mean iWUE (*R* = 0.670, *p* = 0.034), which is mathematically related to Ci. Besides, there was no significant correlation between the mean BERs per leaf surface and the means of A, T, G_H2O_, WUE, SLW and leaf sizes (*p* > 0.2), and there was no significant correlation at all for the mean BERs per leaf dry weight (Table S1). Regarding C‐losses (Figure 3D–F), their means scaled negatively with mean G_H2O_ (*R* = −0.719, *p* = 0.019) and mean LWCs (*R* = −0.738, *p* = 0.015), and positively with mean leaf sizes (*R* = 0.828, *p* = 0.003). Given that the C‐loss is the ratio between BER and A, its negative correlation to G_H2O_ is explained by the strong positive correlation between A and G_H2O_ (Table S1). Similarly, its negative correlation to mean LWC results from the positive correlation of LWC with A and the marginally negative correlation of LWC with BER.

## 4 Discussion

### 4.1 Quality—Intraspecific Variability in the Emission Composition

The emissions of the five major emitted MTs exhibited discrete discontinuous variations in their relative contributions, classifying them into three non‐plastic chemotypes, wherein three of the five compounds clearly co‐varied. Many MT synthases are multiproduct enzymes synthesizing several MT isomers in defined proportions (Degenhardt et al. 2009). Thus, the recurrent discontinuous emission pattern observed in the Cork oak provenances could be due to differences in the activity of only two terpene synthases, one producing predominantly α‐, β‐pinene and sabinene, and one producing predominantly limonene. Myrcene is probably a by‐product of both enzymes, but especially of the limonene‐producing enzyme, as indicated by a moderate positive correlation between the myrcene and limonene fractions of emissions (*R* = 0.55; Figure S4). The activity of each putative MT synthase contributing to the final emission blend of the three chemotypes would depend on genomic, proteomic, but most likely on transcriptomic differences as commonly observed in other studies (Padovan et al. 2017 and references therein).

Numerous studies have documented the diversification of monoterpene production in plant clades, often involving duplication of MT synthase genes, followed by neofunctionalisation due to a few mutations that change the shape of the active site of the enzymes (see e.g., Weng et al. 2012; Christianson 2017; Xu et al. 2021). In this context, the question arises as to what extent the observed chemodiversity in Cork oak is an ancestral trait common with other MT‐emitting oaks and has evolved during species radiations. The phylogeny of oaks has been extensively studied using neutral genetic markers (for review see e.g., Hipp et al. 2020; Kremer and Hipp 2020). According to Denk et al. (2023), the common ancestor of the subgenus *Cerris* originated in northern East Asia and dates to the early Eocene epoch under temperate climates. Subsequently, the sections *Cerris* and *Ilex* split off during migrations. The *Cerris* section expanded north‐western over Siberia, whereas oaks of the section *Ilex* moved south‐westward in subtropical climates of southern China and south‐eastern Tibet, from where they moved further west along the pre‐Himalayan mountains. *Ilex* and *Cerris* oaks joined later in western Eurasia along with some isoprene emitting oaks from eastern North America of the section *Quercus* (e.g., *Q. robur* , *Q. petrea*, *Q. pubescens*).

**FIGURE 4:**
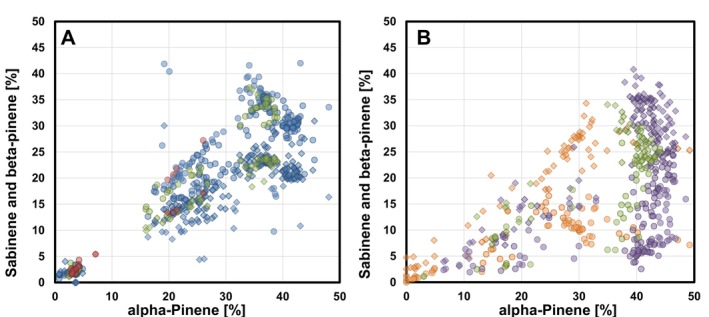
Plots of the proportions of sabinene (circles) and β‐pinene (diamonds) against the proportions of α‐pinene in the emissions of (A) *Q. suber* (Cork oak) and *Q. afares* (African oak) and in the emissions of B: *Q. ilex* (Holm oak) and *Q. coccifera* (Kermes oak). The shown emission data were taken from different studies. In A: Blue: *Q. suber* (this study); green: *Q. suber* (Staudt et al. 2004): Brown: *Q. afares* (Welter et al. 2012). In (B) Purple: *Q. ilex* (Staudt et al. 2001); green: *Q. ilex* (Staudt et al. 2004); red: *Q. coccifera* (Staudt and Visnadi 2023). In all the emissions, Pinene/Sabinene, Limonene and Mixed chemotypes were observed. However, in the emissions from *Q. suber* and *Q. afares*, the proportions of sabinene are higher than those of β‐pinene, whereas in *Q. ilex* and *Q. coccifera*, the opposite is observed.

Regarding the available literature describing the emission composition of MT‐emitting oak species other than cork oak (all in the subgenus *Cerris*; Figure S1), almost all of them report that α‐pinene, sabinene, and β‐pinene are the most important compounds, in proportions similar to those we observed in the Pinene/sabinene chemotype of cork oak. These include the Mediterranean oaks *Q. ilex* and *Q. coccifera* from the section *Ilex*, and *Q. ithaburensis* from the section *Cerris*. In addition, the East Asian oaks *Q. phillyraeoides* (section *Ilex*) as well as *Q. glauca* and *Q. myrsinifolia* (both section *Cyclobalanopsis*). Comparable emission compositions were also found for *Castanopsis sieboldii* and *Castanea crenata* , two species from other Fagaceae genera (Mochizuki et al. 2020). This common feature in the emissions suggests that a Pinene/sabinene chemotype could be the ancestral type within the oak subgenus *Cerris*, which was modified a posteriori during subgenus expansions. Indeed, studies on the variability of VOC emissions within and between populations have identified chemical polymorphisms not only in cork oak (Staudt et al. 2004; Loreto et al. 2009; this study), but also in *Q. ilex* (Staudt et al. 2001, 2004), *Q. coccifera* (Staudt and Visnadi 2023), and *Q. afares* (Welter et al. 2012). All of them express similar Pinene/Sabinene, Limonene, or Mixed chemotypes. However, the proportions of β‐pinene and sabinene produced by putative pinene/sabinene synthases differ between the two oaks of the section *Ilex* ( *Q. ilex* and *Q. coccifera*) and the two oaks of the section *Cerris* ( *Q. suber* and *Q. afares*), but not or less so between oaks of the same section. As can be seen in Figure 4, most individuals of the two *Cerris* oaks exhibit a higher sabinene/β‐pinene ratio than the individuals of the two *Ilex* oaks. Furthermore, differences in the data dispersion among the proportions of α‐, β‐pinene, and sabinene (Figure S5) provide evidence that pinenes together with sabinene are formed by several active enzymes in *Q. ilex* and *Q. coccifera*, but not necessarily in cork oak and *Q. afares*. Finally, more other chemotypes were observed in the emissions of the two *Ilex* oaks, namely a Myrcene chemotype and a 1,8‐Cineol chemotype, which were never observed in the emissions of *Q. afares* and cork oak, as confirmed by the present study. These observations collectively indicate that the MT production in *Q. ilex* and *Q. coccifera* became more diversified than in cork oak.

Indeed, a common result of genetic studies on Mediterranean oak species is that Cork oak has a less diversified gene pool than those of other sympatric oak species and expresses a west–east differentiation pattern in their gene pools with two or a few major clusters (Lumaret et al. 2005; Lumaret and Jabbour‐Zahab 2009; Simeone et al. 2018; Pina‐Martins et al. 2019; López de Heredia et al. 2020; Sousa et al. 2022, 2023; and other references therein). Based on nuclear alloenzyme variation, Toumi and Lumaret (1998) observed a lower genetic diversity in several south‐eastern Cork oak populations including in North Africa than in western Iberian populations, which is consistent with the different degrees of chemical polymorphism we observed in their VOC emissions (Figure 2). With a relatively narrow ecological amplitude, Cork oak has a more restricted and fragmented range than other sympatric oaks such as *Q. ilex* and *Q. coccifera*, which resist better to freezing and snow charge, and grow well on limestone (Toumi and Lumaret 2001; Caudullo et al. 2017). Local bottlenecks and enhanced genetic drifts during repeated retractions and isolations of populations in ice age refugia along with human selection have particularly reduced the genetic diversity in Cork oak. We hypothesize that this genetic erosion is also reflected in its impoverished emission profile compared to *Q. ilex* and *Q. coccifera*. Introgressive hybridization events might have contributed to qualitative and quantitative emission diversifications. Simeone et al. (2018) have described ingressive forms of the non‐emitting oak *Q. cerris* into Cork oak on the Italian peninsula, which may explain the occurrence of non‐emitting cork oaks. With regard to the introgression with *Q. ilex* , in our study, seven Cork oak individuals of the Pinene/sabinene chemotype emitted β‐pinene in higher proportions than sabinene plus relatively large proportions of myrcene, as is typical for the emissions of *Q. ilex* (Figure 4B). Interestingly, all but one of these saplings were from the Var provenance, possibly witnessing a high degree of ancient hybridizations in this population.

### 4.2 Quantity—Intraspecific Variability in BER

To minimize the phenotypic plasticity in MT production, we measured and adapted plants to the same assay conditions using, in the great majority, saplings of the same age that were grown together. Despite this, the BER of the 238 emitting saplings that all released the same five principal MTs varied by up to two orders of magnitude (VC ca 70%). Repeated measurements conducted on the same individuals over the same period revealed only up to fivetimes differences in BER (mean VC ca 40%) indicating that a significant portion of the total between‐plant variability in BER was inherent.

The most striking difference between provenances was the emission‐related losses of assimilated carbon (C‐loss). These were significantly higher in the northern provenances of Maremma, Var, and Catalonia than in the southern provenances of Setubal, Kroumerie, and Sardinia (Table 1). The differences in C‐losses were due to differences in both VOC emissions and photosynthesis. The comparatively low photosynthetic rates of leaves from northern provenances were related to their low stomatal conductance, which was often associated with attributes typical of sclerophylly, including relatively low water contents and Ci values, as well as big leaves and relatively high iWUE and SLW values (Peguero‐Pina et al. 2017; Ghouil et al. 2024). The saplings from the Var provenance showed the most extreme values. These outstanding leaf characteristics of the Var specimens confirm the possibility of strong interspecific gene flow in this population, as already suggested by their unusual emission composition.

Overall, our findings suggest that under common, non‐stress growth conditions, some northern provenances develop more sclerophyllous leaves that invest more in MT production than southern provenances. This may point to different adaptive strategies to cope with local environmental constraints. Big robust leaves resist better to drought and freezing, but are costly to produce, thus entailing a slow return in net carbon gain for plant growth. This investment might be beneficial for seedlings growing in the northern, continental climates at higher elevations exposed to winter freezing and moderate summer drought. By contrast, small fast‐growing leaves with less investment in structural support and protective tissues might be advantageous in populations growing in the southern coastal regions. They optimize the net carbon gain under the mesic and mild winter season and are less costly to replace during severe water deficits. Leaf shedding is an effective way to reduce water losses by transpiration, thus preventing plants from lethal stresses (Staudt et al. 2008; Fallon and Cavender‐Bares 2018).

## 5 Conclusions and Outlook

With a mean BER of ca. 2700 ng m^−2^ s^−1^ consuming more than 2% of the net‐assimilated carbon, our results place Cork oak among the highest MT emitters in the Mediterranean area, rivaling the BERs known for strong isoprene emitters and exceeding those reported for sympatric conifers and aromatic shrubs such as Aleppo pine, Juniper, Thyme, and Rosemary (Owen et al. 2001; Bracho‐Nunez et al. 2013). Nevertheless, few individuals did not release volatile isoprenoids in relevant amounts. Propagating non‐emitting individuals in reforestation programs could provide an opportunity to reduce regional VOC pollution. However, their rarity suggests that they are subject to negative selection. To better understand the underlying reasons, it would be interesting to create half‐sib populations of non‐emitters to elucidate their inheritance mode and to test their ecological and phenological characteristics such as growth rate or vulnerability to abiotic and biotic stresses (e.g., Zuo et al. 2025).

Independent of BER variability, VOC emissions of plants displayed a discontinuous variation in their composition, separating them into three distinct chemotypes. These chemotypes were unevenly distributed across the range, with the two limonene‐producing chemotypes being more common in some western populations and the Pinene/sabinene chemotype being more common in southeastern populations. Comparison with the chemical diversity of emissions reported for other oak species suggests that the Pinene/sabinene chemotype is an ancestral trait common to the subgenus *Cerris*, which was modified during clade radiations, whereby Cork oak emissions became less diversified than the emissions of congeners, possibly due to strong recurrent fragmentation and restrictions of its range and interspecific genetic exchanges.

## Author Contributions

**Michael Staudt:** conceptualization (lead), formal analysis (equal), funding acquisition (lead), investigation (supporting), supervision (lead), writing – original draft (lead), writing – review and editing (lead). **Coralie Rivet:** formal analysis (equal), investigation (lead), writing – original draft (supporting). **Meltem Erdogan:** formal analysis (equal), investigation (lead), writing – original draft (supporting).

## Conflicts of Interest

The authors declare no conflicts of interest.

## Supporting information


**Data S1:** Staudt_etal_Supporting Information Original data.xlsx: Excel sheets with the original data.

**Data S2:** Staudt_etal_Supporting Information Figures S1–S5_TableS1.docx comprising.
**Figure S1:** Overview on the interspecific variability of isoprenoid emissions within the genus *Quercus*.
**Figure S2:** VOC emission rates and composition and photosynthesis rates from four replicate measurements carried out on five Cork oak seedlings during the experimental period.
**Figure S3:** Dendrogram from hierarchical cluster analysis and observation diagram from factorial discriminant analysis showing that the variation in the composition of the five major monoterpenes emitted by 238 Cork oak individuals can be categorized into three different chemotypes.
**Figure S4:** Plot of the proportions of α‐pinene versus the proportions of the four other main MTs in the emissions from 238 Cork oak saplings.
**Table S1:** Matrix with results of Pearson correlation among the provenance mean values of the basal emission rate, relative leaf water content, leaf size, specific leaf weight, leaf internal CO_2_ concentration, photosynthesis, water vapor conductance, water use efficiency, intrinsic water use efficiency and the % fraction of assimilated carbon lost by VOC emission.
**Figure S5:** Plots of the proportions of α‐pinene against the proportions of β‐pinene, sabinene and the sum of β‐pinene plus sabinene in the emissions of four different oaks species.

## Data Availability

The original data are actually available as supporting information—S1 of the article (Staudt_etal_Supporting Information Original data.xlsx).
