# Supplementary material for: Diversity of Volatile Emissions From Cork Oak: Quantity and Quality Vary Independently Across Its Range
Source: Ecol Evol. 2025 Aug 31;15(9):e72093. doi: 10.1002/ece3.72093 (PMC12399320; doi:10.1002/ece3.72093)
Supplement: Supplementary file 2 — Data S2: Staudt_etal_Supporting Information Figures S1–S5_TableS1.docx comprising. Figure S1: Overview on the interspecific variability of isoprenoid emissions within the genus Quercus. Figure S2: VOC emission rates and composition and photosynthesis rates from four replicate measurements carried out on five Cork oak seedlings during the experimental period. Figure S3: Dendrogram from hierarchical cluster analysis and observation diagram from factorial discriminant analysis showing that the variation in the composition of the five major monoterpenes emitted by 238 Cork oak individuals can be categorized into three different chemotypes. Figure S4: Plot of the proportions of α‐pinene versus the proportions of the four other main MTs in the emissions from 238 Cork oak saplings. Table S1: Matrix with results of Pearson correlation among the provenance mean values of the basal emission rate, relative leaf water content, leaf size, specific leaf weight, leaf internal CO2 concentration, photosynthesis, water vapor conductance, water use efficiency, intrinsic water use efficiency and the % fraction of assimilated carbon lost by VOC emission. Figure S5: Plots of the proportions of α‐pinene against the proportions of β‐pinene, sabinene and the sum of β‐pinene plus sabinene in the emissions of four different oaks species. [file ECE3-15-e72093-s001.docx]

Supporting Information Figs S1 – S5 and Table S1

Diversity of volatile emissions from cork oak: quantity and quality vary independently across its range

**Michael Staudt*, Coralie Rivet, Meltem Erdogan**

CEFE, CNRS, EPHE, IRD, Univ Montpellier, Montpellier, France

*For correspondence: michael.staudt@cefe.cnrs.fr

**Figure S1** (see next page). Interspecific variability of isoprenoid emissions within the genus ***Quercus***. The shown oak phylogeny was adapted from Denk et al. (2017), after which the genus is subdivided into the two subspecies (***Cerris*, *Quercus***) comprising respectively three (*Cyclobalanopsis*, *Ilex*, *Cerris*) and five sections (*Lobatae*, *Protobalanus*, *Ponticae*, *Virentes*, *Quercus*). The approximate number of species within each section is given in brackets. For the sections that have only a few species (*Cerris*, *Protobalanus*, *Ponticae*, *Virente*), all species have been listed, including those for which we could not find information on their constitutive VOC emissions. VOC emission data were compiled considering the following publications (and other references therein):

Bertin et al. 1997; doi: 10.1016/S1352-2310(97)00080-0

Steinbrecher et al., 1997, doi: 10.1016/S1352-2310(97)00076-9)

Loreto et al., 1998; doi: 10.1007/s004420050520

Csiky and Seufert, 1999; doi: 10.1890/1051-0761(1999)009[1138:TEOMOA]2.0.CO;2

Kesselmeier and Staudt, 1999; doi: 10.1023/A:1006127516791

Harley et al., 1999; doi: 10.1007/s004420050709

Guenther et al., 1999; 10.1016/S1464-1909(99)00062-3

Loreto, 2002; doi: 10.1078/1433-8319-00033

Lim et al., 2011; doi: 10.1016/j.atmosenv.2011.01.066

Okumura et al., 2008; doi: 10.2525/ecb.46.257

Tani and Kawawata, 2008; doi: 10.1016/j.atmosenv.2008.01.059

Tani et al., 2011; doi: 10.1016/j.atmosenv.2011.08.003

Steinbrecher et al., 2009; doi: 10.1016/j.atmosenv.2008.09.072

Welter et al., 2012; doi: 10.1093/treephys/tps069

Monson et al., 2013; doi: 10.1111/pce.12015

Yaman et al. 2015; doi: 10.4209/aaqr.2014.04.0082

Mochizuki et al., 2020; doi: 10.1080/13416979.2020.1779425

Bao et al., 2023; doi: 10.1016/j.envpol.2022.120886

Staudt et al., 2023; doi: 10.1525/elementa.2023.00043

Baek et al., 2024; doi: 10.1016/j.atmosenv.2024.120654

Tani et al., 2024; doi: 10.1186/s40645-024-00645-8

Yu et al., 2024; doi: 10.1016/j.atmosenv.2023.120238

Li et al., 2025; doi: 10.1021/acs.est.5c01132


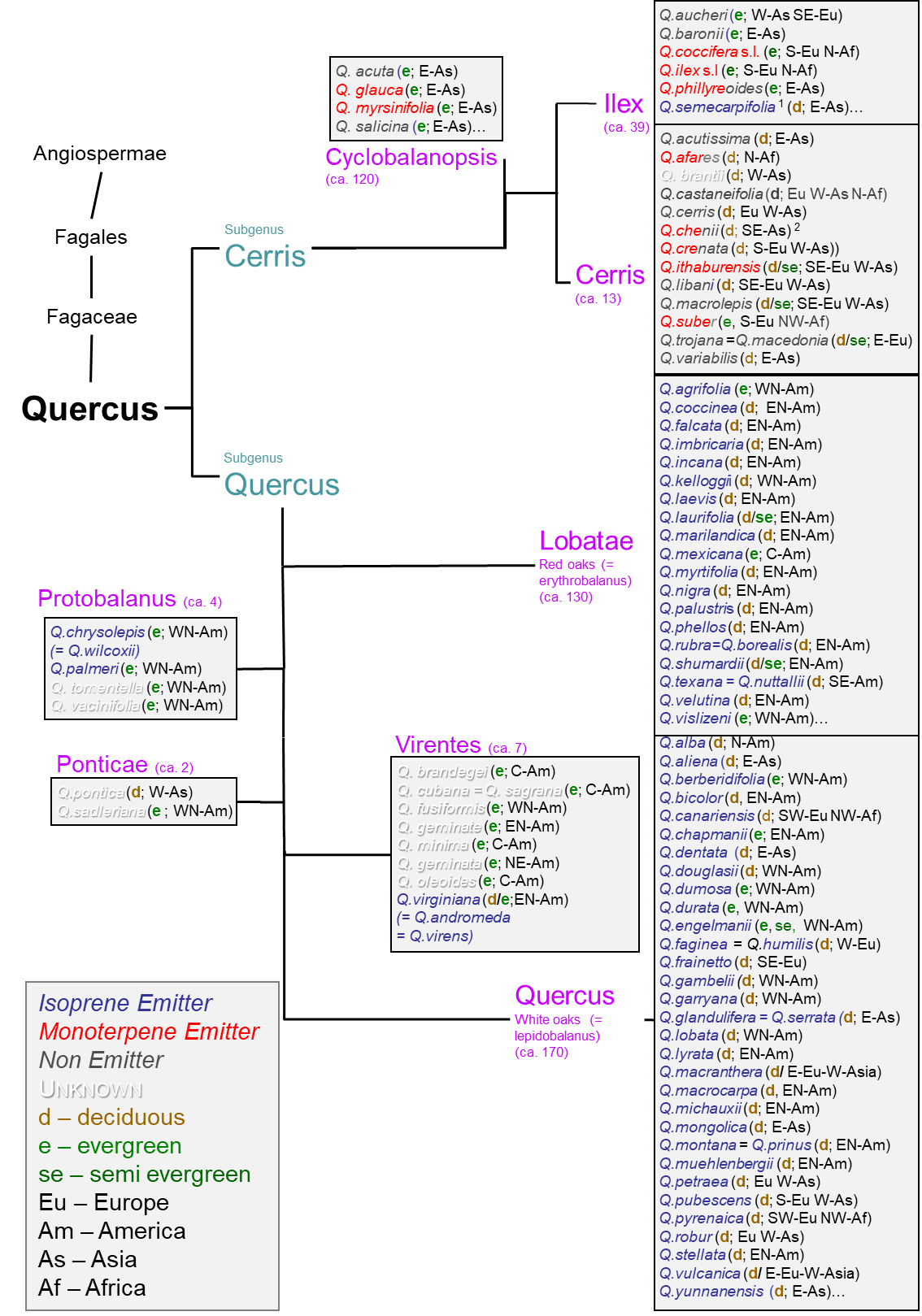


^1^ based on a single study by Loreto et al. (1998), which measured VOC emissions from an unknown number of seedlings grown from acorns collected in the field.

^2^ classified as a non-isoprene emitter in Harley et al. (1999), without giving details on whether this species emits monoterpenes or not.

**Figure S2.** Repeated measurements of leaf VOC emissions and photosynthesis from five cork oak saplings originating from the provenances Setubal, Catalonia, Var, Sardinia and Maremma. (A) Emission rate of the sum of the five main VOCs (red columns) and photosynthesis rate (green columns). (B) Relative proportions of the five main VOCs. During the experimental period, four replicate measurements were carried out on each sapling at 30 °C and approx. 1500 µmol m^-2^ s^-1^ incident PPFD on annual leaves of different twigs. The results show that the compositional fingerprint of the VOC production of each tree is quite stable, while the quantities emitted are subject to greater fluctuations.


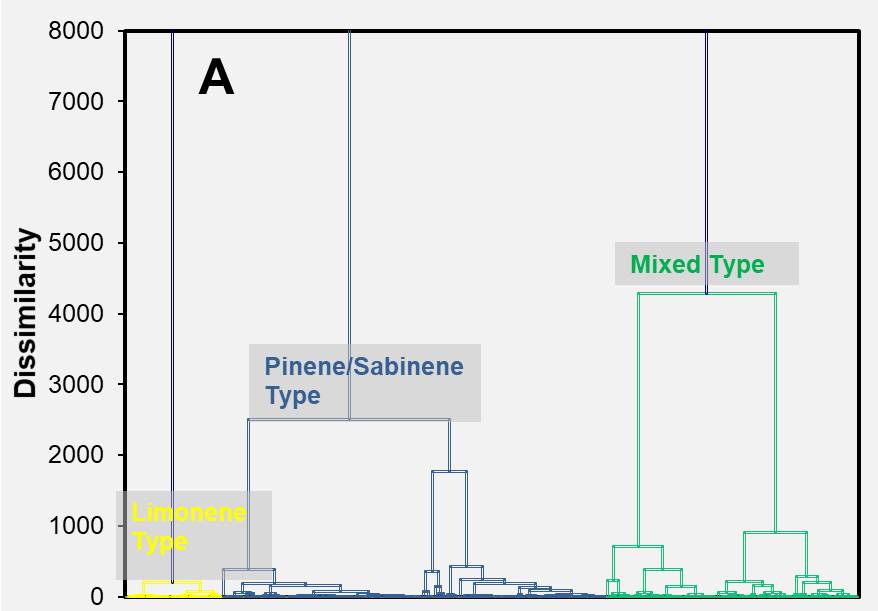

**Figure S3.** The compositional variation of the 5 major monoterpenes emitted from 238 cork oak individuals clusters in three distinct chemotypes: Limonene type (13 %, yellow), Pinene/Sabinene type (52.5 %, blue) and Mixed type (34,5 %, green). Panel A shows the dendrogram obtained from hierarchical cluster analysis using Ward’s technique. Panel B shows the observation plot resulting from Factorial Discriminant Analyses (Centroids in red). More than 99 % of the observed variance is represented by Factor 1 (x-axis) correlated with the proportions of limonene, pinenes and sabinene (small inserted graph).


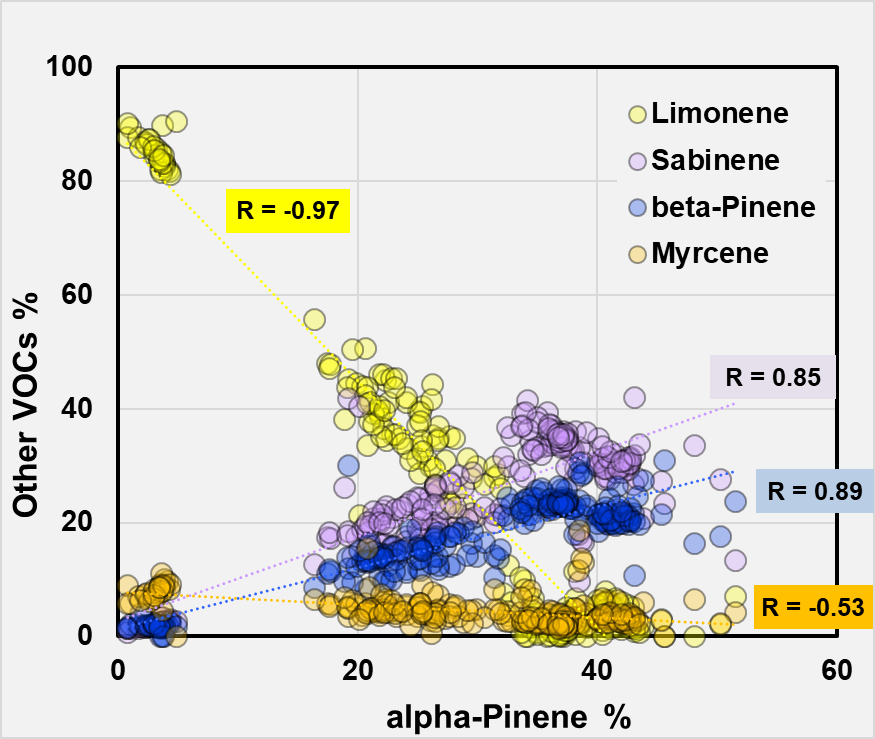


**Figure S4.** Plot of the proportions of α-pinene against the proportions of the four other major VOCs emitted from 238 Cork oak saplings (sum of 5 = 100 %). Dotted lines show best-fit correlations with correlation coefficients R assuming linear relationships. Proportions (fractions) are mathematically negatively correlated. If the absolute rates of the five VOCs all varied randomly, the resulting correlations between the individual components would be insignificant with R-values < -0.3 in most cases. Therefore, the strong positive correlations between the proportions of pinenes and sabinene in the emissions (R = 0.85 to 0.89) reveal that these three monoterpenes are always synthesised and emitted together in cork oak leaves, while limonene is apparently produced independently from pinenes and sabinene (R = -0.94 to -0.97). Myrcene, which is only emitted in small amounts, could be predominantly a by-product of limonene synthesis (R = 0.55).

**Table S1.** Matrices with results of Pearson correlation among the provenance mean values of the basal emission rate per leaf surface area (BER), relative leaf water content (LWC), leaf size, specific leaf weight (SLW), leaf internal CO_2_ concentration (Ci), photosynthesis (A), water vapour conductance (G_H2O_), water use efficiency (WUE), intrinsic water use efficiency (iWUE) and fraction of assimilated carbon lost by VOC emission (C-loss). The upper table shows the correlation coefficients (*R*) and the lower table the corresponding *P*-values. Values in **bold** are significant at alpha = 0.05. Values in red denote correlations between two mathematically dependent variables, which should therefore be interpreted with caution.

| Variable | BER^1^ |  | LWC | Leaf size | SLW | Ci | A | GH2O | WUE | iWUE | C-loss |
| --- | --- | --- | --- | --- | --- | --- | --- | --- | --- | --- | --- |
| BER^1^ | **0** |  | 0.072 | 0.220 | 0.205 | **0.003** | 0.914 | 0.386 | 0.500 | **0.034** | 0.285 |
| LWC | 0.072 |  | **0** | 0.095 | 0.253 | 0.134 | **0.011** | **0.007** | 0.469 | **0.033** | **0.015** |
| Leaf size | 0.220 |  | 0.095 | **0** | 0.821 | 0.522 | 0.167 | 0.308 | 0.902 | 0.509 | **0.003** |
| SLW | 0.205 |  | 0.253 | 0.821 | **0** | 0.059 | 0.998 | 0.617 | 0.235 | 0.160 | 0.828 |
| Ci | **0.003** |  | 0.134 | 0.522 | 0.059 | **0** | 0.678 | 0.140 | 0.671 | **0.000** | 0.304 |
| A | 0.914 |  | **0.011** | 0.167 | 0.998 | 0.678 | **0** | **0.000** | 0.061 | 0.136 | **0.007** |
| G_H2O_ | 0.386 |  | **0.007** | 0.308 | 0.617 | 0.140 | **0.000** | **0** | 0.114 | **0.004** | **0.019** |
| WUE | 0.500 |  | 0.469 | 0.902 | 0.235 | 0.671 | 0.061 | 0.114 | **0** | 0.547 | 0.505 |
| iWUE | **0.034** |  | **0.033** | 0.509 | 0.160 | **0.000** | 0.136 | **0.004** | 0.547 | **0** | 0.120 |
| C-loss | 0.285 |  | **0.015** | **0.003** | 0.828 | 0.304 | **0.007** | **0.019** | 0.505 | 0.120 | **0** |

^1^ The correlations between mean BERs per leaf dry mass with other variables were not significant (Ci: *R* = -0.612, *P* = 0.060; iWUE: *R* = 0.513, *P* = 0.137; LWC: *R* = -0.477, *P* = 0.163).

**Figure S5.** Plot of the proportions of α-pinene against the proportions of β-pinene (blue dots), sabinene (pink dots) and the sum of both β-pinene plus sabinene (grey dots) in the emissions of different oaks species. A: Cork oak (*Q. suber*), this study; B: Cork oak, Staudt et al. (2004); C: African oak (*Q. afares*), Welter et al., (2012); D: Holm oak (*Q. ilex*), Staudt et al. (1999); E: Holm oak (*Q. ilex*), Staudt et al. (2004); F: Kermes oak (*Q. coccifera*), Staudt and Visnadi (2023). Current oak phylogeny classifies Cork oak and African oak in the section *Cerris*, and Kermes oak and Holm oak in the section *Ilex*, both of which belong to the oak subspecies *Cerris* (Fig. S1). The VOC emissions of all oak species contain the same five major monoterpenes (sum = 100 %), the proportions of which vary within each species in similar chemotypes. The positive correlations between the proportions of α-pinene, β-pinene and sabinene indicates that all three compounds are synthesized jointly, possibly by multiproduct enzymes (see also Fig. S4). However, while in the emissions of Holm and Kermes oak the correlation fit to α-pinene is better for the sum of β-pinene and sabinene than for the single compounds, it is more or less the same in the emissions of Cork and African oak. This indicates that the three compounds are produced by a single enzyme in *Cerris* oaks, but by two or more enzymes in *Ilex* oaks (see also Staudt and Visnadi (2023) for further evidence).
